# A perspective of *Genes and Environment* for the development of environmental mutagen research in Asia

**DOI:** 10.1186/s41021-017-0083-y

**Published:** 2017-10-01

**Authors:** Takashi Yagi

**Affiliations:** Laboratory of Molecular and Cellular Genetics, Department of Biological Sciences, Graduate School of Science, Osaka Prefecture University, 1-2 Gakuen-cho, Naka-ku, Sakai, Osaka, 599-8570 Japan

**Keywords:** Japanese Environmental Mutagen Society, Asian Association of the Environmental Mutagen Societies, *Genes and Environment*, Genotoxicity, Mutagenesis

## Abstract

Two years have passed since the Japanese Environmental Society (JEMS) made the official journal *Genes and Environment* (G&E) open access. Current subjects on environmental mutagen research to further advance this field are described herein, and the roles of JEMS and G&E are discussed. Various important subjects are being investigated in current research fields such as severe environmental pollution in Asian countries; the identification of new hazardous substances and elucidation of mutation mechanisms using newly developed techniques; the development of new genotoxicity assays including in silico predictions using information technology and artificial intelligence as well as bioassays. International exchange by scientists is important for advancing these research fields through international conferences such as the 12th International Conference and 5th Asian Congress on Environmental Mutagens and the 7th International Workshop on Genotoxicity Testing that will be held in 2017. G&E provides a common platform for high quality environmental mutagen research, contributes to the dissemination of Asian environmental mutagen research, and potentiates the level of research being conducted.

## Background

Environmental mutagens are defined as chemical and physical agents in the environment that induce genetic mutations or increase mutation rates during the human life span [1]. Most mutagens act as human carcinogens or exert genotoxic effects on the next generation via germ cells.

The Japanese Environmental Mutagen Society (JEMS) was founded in 1972 in order to promote basic and applied research on mutagens. JEMS also aims to spread their reference information and technology to the general population [2]. The first president of JEMS was Dr. Takashi Sugimura, the National Cancer Center. JEMS research includes searches for unidentified mutagenic substances in foods, water, and the atmosphere; the measurement of mutagenic substances in the environment; the elucidation of mutagenic and carcinogenic mechanisms; the elucidation of differences in genetic susceptibility among humans or animals; investigations on the complex effects of some mutagens; genotoxicity tests on foods, medicine, and chemical materials; the development and modification of genotoxicity tests; and carcinogenic risk evaluations of substances. JEMS also contributes to government plans such as assessing safety standards for chemical issues and establishing standard genotoxicity tests. JEMS has a long history of these studies and many eminent achievements, which contribute to the promotion of human health [2].

JEMS has been one of the core members of the International Association of Environmental Mutagen Societies (IAEMS) since it was founded in 1973 during the 1st International Conference on Environmental Mutagens (ICEM) held by Dr. Bruce Ames in Asilomar, USA [3]. At this conference, many substances including known carcinogens were identified as mutagens. These findings had an impact on research on carcinogenicity worldwide [4].

JEMS has published an official journal, *Environmental Mutagen Research*, since 1973, and the journal then moved to *Genes and Environment* (G&E) in 2006. The society subsequently published G&E quarterly and successfully transmitted their contents to the international scientific community. Special issues of G&E are useful and convenient sources for highlighting the timely topics of environmental mutagenesis and genotoxicology, such as nanomaterials, air pollution, radiation risks, genotoxic thresholds, and epigenetics [3]. In 2015, JEMS decided to make the journal open access, and has published it for two years.

Current and future important subjects related to environmental mutagen research that further advances this research field are described herein, and the roles of JEMS and G&E for this purpose are discussed.

## Environmental issues in Asia

Many Asian countries are now facing various issues associated with environmental pollution mainly due to rapid economic expansion. Increases in the risk of lung cancer caused by suspended particulate matter (SPM), particularly that smaller than 2.5 μm (PM2.5) in large Asian cities, and air pollutants crossing national borders still continue [5, 6]. PM2.5 contains various types of carcinogenic polyaromatic hydrocarbons. Soil is also contaminated by air pollutants in rain. Groundwater polluted with arsenic and mercury is a health hazard to Asian populations [7, 8]. Many rivers in large Asian cities are polluted with chemicals mainly due to insufficient sewage treatment systems. Local water management offices report that even major rivers such as the Ganges in Kolkata, Chao Phraya in Bangkok, Song Hong in Hanoi, Huangpu in Shanghai, and Hangang in Seoul have a biological oxygen demand (BOD), a measure of organic substance pollution, of greater than 3.5 mg/L. The identification and measurement of environmental substances hazardous to humans and the ecosystem remain important issues and novel challenges. Unknown toxic and mutagenic substances may be present in the Asian environment. Therefore, before the general population is heavily exposed to these substances, we need to estimate their health risks and identify the sources of pollutants.

## New techniques for mutation research

DNA damage induction, DNA repair and DNA replication errors are mainstream subjects for studying the mechanisms of mutagenesis. The development of a very high sensitivity liquid chromatography-tandem mass spectrometric analysis (HS-LC/MS/MS) allows trace amounts of toxic materials to be detected in environmental samples. This HS-LC/MS/MS also measures small amounts of DNA adducts, and may be applied to an adductome analysis that comprehensively detects adducts in the human genome [9, 10].

In DNA repair and replication research, the use of mutant human cells is a common practice for investigating the functions of genes and proteins. Cells deficient in DNA repair genes and translesion DNA polymerase genes have been obtained from patients with hereditary diseases such as classical and variant xeroderma pigmentosum, Cockayne syndrome, and ataxia telangiectasia. Alternatively, an RNA interference (RNAi) technique has commonly been used to suppress the functions of these genes artificially in cells [11]; however, the new CRISPR/Cas9 technique called genome editing can cause mutations in the target genes of interest [12], and will lead to advances in mutation research.

The new generation DNA sequencer (NGS) greatly contributes to mutation research due to a decrease in the price of the whole genome sequencing service. NGS may detect mutations in the whole genome of cells without selection. We now have the ability to compare genome mutations in individuals living in high cancer incidence areas with those in a reference area. Genome mutations in animals exposed to a particular mutagen provide important data for understanding chemical carcinogenesis [13].

In order to elucidate the mechanisms underlying chemical carcinogenesis, transcription abnormalities that may be caused by epigenetic changes, transcription errors due to translesion RNA synthesis, and the decay of mRNA surveillance mechanisms are also important in the field of mutation research.

These new techniques will result in great advances in environmental mutagen research.

## Development of new genotoxicity tests

Various bioassays to detect genotoxicity have been developed and used to guarantee the safety of new substances in the fields of pharmaceutical screening and functional material development: e.g., the Ames test, in vitro and in vivo micronucleus tests, chromosome aberration test, *hprt* and *tk* mutation assays, comet assay, rec-assay, umu test, and mutation reporter-transgenic animals. The Pig-a assay and γH2AX test are currently being considered for validation as new bioassays, which are important in the field of genotoxicity testing and regulatory sciences [14, 15].

The prediction of genotoxic activities based on their chemical structures using information technology (IT) is currently in progress for the development of new pharmaceuticals, and has already been applied to the genotoxic impurities of new pharmaceuticals [16]. The development of HS-LC/MS/MS has resulted in advances in genomic adductome analyses on target organs from animals treated with pharmaceuticals. NGS has the ability to confirm whether pharmaceuticals induce mutations in animals using a whole genome analysis. The combination of these methods may replace traditional genotoxicity bioassays.

## Importance of regulatory sciences

JEMS has contributed to the establishment of testing guidelines for genotoxic evaluations of substances, such as food additives, pesticides, and industrial chemicals [17]. Most new substances are evaluated according to these guidelines, which have been indispensable for hazard identification and risk assessments. These regulatory sciences will contribute to the establishment of standard limits for new chemicals in foods, effluent water, and the environment in Asian countries.

Risk assessments of carcinogens lacking mutagenicity such as aneugens and tumor promoters have been discussed. Evaluations of nucleic acid medicine, antibody medicine, and regenerative medicine represent future issues for regulatory sciences. Since environmental mutagen research started in the early 1970s, large amounts of data regarding toxicity and the action mechanisms of each substance have accumulated in many journals; therefore, the development of a large data analysis using IT or artificial intelligence (AI) without the need for manual work will be required for rapid and accurate risk assessments.

Some of these recent regulatory issues will be discussed at the 7th International Workshop on Genotoxicity Testing (IWGT) in Tokyo from November 8–10, 2017, which will be held after the 46th JEMS Annual Meeting [18].

## Cooperation through international conferences

JEMS is a member of the International Association of the Environmental Mutagenesis and Genomics Societies (IAEMGS) and the Asian Association of the Environmental Mutagen Societies (AAEMS). IAEMGS was previously named IAEMS but the name was changed in 2013 to embrace a wider range of research on genome safety such as genomics and epigenomics. IAEMGS and AAEMS will have a joint international conference, the 12th International Conference and 5th Asian Congress on Environmental Mutagens (ICEM-ACEM 2017) in Incheon, Korea from November 12 to 16, 2017 [19]. JEMS organized ICEM in Tokyo and Shizuoka in 1981 and 2001, respectively, and ACEM at Kita-Kyushu in 2007. G&E will invite review articles from ICEM-ACEM 2017 plenary and symposium speakers who present leading research topics on environmental mutagenesis. JEMS will place the G&E presentation booth at the conference hall and transmit the contents of the journal to participants. At the booth, JEMS and participants will discuss further developments in environmental mutagen research and related research areas as well as the role of G&E for this purpose.

## Roles of *Genes and Environment*

JEMS will receive a Grant-in-Aid for Scientific Research, the category of which is “enhancement of international dissemination of information”, for five years from 2017, and this grant is awarded by the Japan Society for Promotion of Science (JSPS) [20], an administrative institution under the Ministry of Education, Culture, Sports, Science and Technology, Japan. The aim of the grant to JEMS is “the dissemination of Asian environmental mutagen research from Japan”. JEMS cooperates with Asian scientists in the field of environmental mutagens, and attempts to disseminate various types of distinguished research performed worldwide, particularly in Asia. G&E assists in the publishing of research findings from the member societies of AAEMS. G&E invites Asian scientists to the Editorial Board to cooperate with them for a peer review of submitted papers. G&E publishes papers with open access and a CC-BY Creative Commons License [21], and, thus, provides a common platform to share information among scientists.

G&E makes efforts to be indexed in the journal citation database, Web of Science, in order to obtain an Impact Factor (IF) because many Asian scientists consider studies published in journals possessing IF to be highly esteemed. This is called the myth of IF. G&E has been indexed in PubMed, PubMed Central, DOAJ, and Scopus. Scopus also publishes a similar impact value, the CiteScore [22]. The CiteScore of G&E was 1.74 in 2016, which indicates that articles published in G&E between 2013 and 2015 were cited 1.74 times per article on average in 2016. JEMS continuously makes efforts to increase citation rates.

## Conclusions

In order to further advance environmental mutagen research, scientists in this field always attempt to adopt new techniques, as discussed above, particularly analytical instruments, the techniques of molecular and cellular biology, and those using IT and AI, which will open new research areas in the field. G&E welcomes these studies in order to achieve breakthroughs in environmental mutagen research.

Various unsolved environmental issues exist in Asia and become the subjects of research. In order to perform research at a higher level, international cooperation to exchange mutually strong areas is more significant than competition. Scientific discussion at international conferences such as ICEM-ACEM 2017 and IWGT 2017 in Korea and Tokyo, respectively, will assist cooperative research in Asia. G&E continuously supports the dissemination of high quality environmental mutagen research and potentiates the level of research being performed in Asia. JEMS expects G&E to become a common platform to discuss environmental research in Asia.

## Abbreviations

AAEMS: The Asian Association of the Environmental Mutagen Societies
AI: Artificial intelligence
BOD: Biological oxygen demand
G&E: *Genes and Environment*
HS-LC/MS/MS: High sensitivity liquid chromatography-tandem mass spectrometric analysis
IAEMGS: The International Association of the Environmental Mutagenesis and Genomics Societies
IAEMS: The International Association of Environmental Mutagen Societies
ICEM: The International Conference on Environmental Mutagens
ICEM-ACEM 2017: The 12th International Conference and 5th Asian Congress on Environmental Mutagens
IF: Impact factor
IT: Information technology
IWGT: The International Workshops on Genotoxicity Testing
JEMS: The Japanese Environmental Mutagen Society
JSPS: Japan Society for Promotion of Science
NGS: New generation DNA sequencer
RNAi: RNA interference
SPM: Suspended particulate matter
